# An Italian Multicenter Study on Anti-NXP2 Antibodies: Clinical and Serological Associations

**DOI:** 10.1007/s12016-021-08920-y

**Published:** 2022-01-29

**Authors:** Micaela Fredi, Ilaria Cavazzana, Angela Ceribelli, Lorenzo Cavagna, Simone Barsotti, Elena Bartoloni, Maurizio Benucci, Ludovico De Stefano, Andrea Doria, Giacomo Emmi, Martina Fabris, Marco Fornaro, Federica Furini, Maria Grazia Giudizi, Marcello Govoni, Anna Ghirardello, Luca Iaccarino, Fiorenzo Iannone, Maria Infantino, Natasa Isailovic, Maria Grazia Lazzaroni, Mariangela Manfredi, Alessandro Mathieu, Emiliano Marasco, Paola Migliorini, Carlomaurizio Montecucco, Boaz Palterer, Paola Parronchi, Matteo Piga, Federico Pratesi, Valeria Riccieri, Carlo Selmi, Marilina Tampoia, Alessandra Tripoli, Giovanni Zanframundo, Antonella Radice, Roberto Gerli, Franco Franceschini, FIRMA (Forum Italiano per la Ricerca nelle Malattie Autoimmuni)

**Affiliations:** 1 Rheumatology and Clinical Immunology Unit, ASST Spedali Civili and Department of Clinical and Experimental Sciences, University of Brescia, Brescia, Italy; 2 Division of Rheumatology and Clinical Immunology, Humanitas Clinical and Research Center - IRCCS, Rozzano (Mi), Italy; 3 Department of Biomedical Sciences, Humanitas University, Rozzano (Mi), Italy; 4 Division of Rheumatology, University and IRCCS Policlinico S. Matteo, Pavia, Italy; 5 Division of Rheumatology, Department of Clinical and Experimental Medicine, University of Pisa, Pisa, Italy; 6 Rheumatology Unit, Department of Medicine, University of Perugia, Perugia, Italy; 7 Immunology and Allergy Laboratory Unit S. Giovanni Di Dio Hospital, Azienda USL Toscana Centro, Firenze, Italy; 8 Rheumatology Unit, Department of Medicine, University of Padova, Padova, Italy; 9 Department of Experimental and Clinical Medicine, University of Firenze, Firenze, Italy; 10 Institute of Clinical Pathology, Department of Laboratory Medicine, University Hospital of Udine, Udine, Italy; 11 Unit of Rheumatology, Department of Emergency and Organ Transplantation, University of Bari, Bari, Italy; 12 Department of Medical Sciences, Rheumatology Unit, University of Ferrara and Azienda Ospedaliero-Universitaria S. Anna, Cona (Ferrara), Italy; 13 Rheumatology Unit, Department of Medical Sciences and Public Health, University of Cagliari and AOU University Clinic, Cagliari, Italy; 14 Clinical Immunology Unit, Department of Clinical and Experimental Medicine, University of Pisa, Pisa, Italy; 15 Department of Rheumatology, Sapienza-University of Rome, Rome, Italy; 16 Clinical Pathology Unit, Department of Biomedical Sciences and Human Oncology, University “Aldo Moro” of Bari, Bari, Italy; 17 Department of Microbiology and Virology, ASST Santi Paolo E Carlo, San Carlo Hospital, Milan, Italy

**Keywords:** Anti-NXP2, Immunoprecipitation (IP), Line blot (LB), Dermatomyositis (DM), Myositis, Autoantibodies

## Abstract

The identification of anti-NXP2 antibodies is considered a serological marker of dermatomyositis (DM), with calcinosis, severe myositis and, in some reports, with cancer. Historically, these associations with anti-NXP2 antibodies have been detected by immunoprecipitation (IP), but in the last few years commercial immunoblotting assays have been released. The aim of this collaborative project was to analyse the clinical features associated to anti-NXP2 antibodies, both with commercial line blot (LB) and IP. Myositis-specific and myositis-associated autoantibodies were detected in single centres by commercial line blot (LB); available sera were evaluated in a single centre by protein and RNA immunoprecipitation (IP), and IP-Western blot. Sixty patients anti-NXP2+ (NXP2+) positive by LB were compared with 211 patients anti-NXP2 negative with idiopathic inflammatory myositis (IIM). NXP2+ showed a younger age at IIM onset (*p* = 0.0014), more frequent diagnosis of dermatomyositis (*p* = 0.026) and inclusion-body myositis (*p* = 0.009), and lower rate of anti-synthetase syndrome (*p* < 0.0001). As for clinical features, NXP2+ more frequently develop specific skin manifestations and less frequently features related with overlap myositis and anti-synthetase syndrome. IP confirmed NXP2 positivity in 31 of 52 available sera (62%). Most clinical associations were confirmed comparing NXP2 LB+/IP+ versus NXP2-negative myositis, with the following exceptions: inclusion-body myositis diagnosis was not detected, whilst dysphagia and myositis were found more frequently in NXP2 LB+/IP+ patients. The 21 LB+ /IP-myositis patients did not show differences in clinical features when compared with the NXP2-myositis patients and more frequently displayed multiple positivity at LB. Risk of developing cancer-associated myositis was similar between NXP2-positive and NXP2-negative myositis patients, either when detected by LB or IP. Protein-IP confirmed NXP2 antibodies in nearly 60% of sera positive for the same specificity with commercial assay. Double-positive cases rarely occurred in myositis patients with a clinical diagnosis other than dermatomyositis. Patients only positive by LB (LB+/IP−) did not display clinical features typical of NXP2. NXP2 positivity by LB should be confirmed by other methods in order to correctly diagnose and characterize patients affected by idiopathic inflammatory myositis.

**Supplementary Information:**

The online version contains supplementary material available at 10.1007/s12016-021-08920-y.

## Introduction

Idiopathic inflammatory myopathies (IIM) are a group of heterogeneous autoimmune inflammatory diseases primarily involving muscle and skin [1]. IIM subtypes include dermatomyositis (DM), polymyositis (PM), anti-synthetase syndrome (ASS), overlap myositis (OM), immune-mediated necrotising myopathy (IMNM) and sporadic inclusion-body myositis (IBM) [1]. Approximately 60–65% of IIM patients have detectable serum autoantibodies, namely myositis-specific autoantibodies (MSA), which are unique to IIM and usually mutually exclusive to one another, or myositis-associated autoantibodies (MAA) which can occur in other connective tissue diseases (CTD) or be present along with MSA [2]. Although only anti-Jo-1 antibodies have been included in the recent EULAR/ACR classification criteria for IIM [3], it was acknowledged that several other MSA also carry clinical value, due to their ability to stratify specific disease subsets [2, 4].

Anti-NXP2 antibodies, originally described in juvenile DM [5], were subsequently identified and confirmed as significantly associated with adult DM [6]. Anti-NXP2 antibodies recognize a 140-kDa nuclear protein (also known as MORC3), involved in transcriptional regulation [7, 8], localized in promyelocytic (PML) bodies in nucleoplasm, resulting in a multiple nuclear dot-like pattern on the indirect immunofluorescence on HEp-2 cells (AC-6 according to the International Consensus of ANA Patterns (ICAP)) [9]. The clinical phenotype associated with anti-NXP2 antibodies is characterized by DM skin rash, calcinosis, periorbital oedema, severe myositis and dysphagia [2, 6, 8, 10–12], whilst controversial data regarding cancer association were reported [6, 12–15]. Historically, these clinical associations have been described when the immunoprecipitation (IP) technique was used for anti-NXP2 detection. Commercial line/dot immunoassays have been released in the last few years; to date, the performance of these tests in IIM is still under discussion [16–26].

The aim of this collaborative project was to analyse the clinical features associated with anti-NXP2 antibodies in a large Italian IIM cohort, including the occurrence of concomitant cancer, using both commercial line blot (LB) and homemade IP.

## Materials and Methods

### Patients

This study was conducted in the frame of the FIRMA group (Forum Interdisciplinare per la Ricerca nelle Malattie Autoimmuni). This project started in 2018 with the aim to collect clinical and serological data from several Italian centres belonging to the FIRMA group: 13 centres collected data on 61 patients locally positive for anti-NXP2 antibodies (NXP2+) by LB.

As control group we collected the same data on 211 patients with a diagnosis of IIM negative for anti-NXP2 (NXP2−) followed-up by two third-level centres (Brescia and Pavia University).

DM, PM and OM were defined according to Bohan and Peter Criteria [27], whereas ASS [28], IBM [29] and IMNM [30] were diagnosed according to the currently used definitions.

Clinical data were obtained from clinical charts. Disease onset was considered as the first skin, joint, muscle or constitutional symptom/sign related to IIM. For all the patients, demographic and epidemiological data, extramuscular findings, including skin manifestations (heliotrope rash, Gottron’s papule, mechanic’s hands, sclerodactyly, cutaneous ulcerations), calcinosis, arthritis, Raynaud’s phenomenon, dysphagia and myocarditis were collected. Muscle involvement was defined when patients presented at least one condition amongst muscle enzymes’ elevation, muscle weakness, presence of typical electromyography (EMG) alterations and/or inflammatory findings on muscle biopsy. Interstitial lung disease (ILD) was defined by high-resolution computed tomography (HRCT) chest scan. Finally, data regarding the presence of a neoplastic disease during patient’s life were retrieved: cancer-associated myositis (CAM) was defined as neoplastic disease onset 3 years before or after myositis’ diagnosis, according to Yang et al. [31].

### Autoantibody Detection

Data related to MSA and/or MAA identification were collected from clinical charts. MSA and MAA were detected in single centres by commercial LB (Autoimmune Inflammatory Myopathy profile 16 antigens OJ, EJ, PL-12, PL-7, SRP, Jo-1, Ro52, PM/Scl-75, PM/Scl-100, Ku, SAE1, NXP2, MDA5, TIF1-gamma, Mi-alpha, Mi-2beta; Euroimmun, Germany). The cut-off value for autoantibody positivity was set by the manufacturer at 11 AU, whereas 5–10 AU are considered borderline as indicated by the manufacturer. Fifty-two anti-NXP2+ sera were available for IP assay, as confirmatory anti-NXP2 test, and for anti-nuclear antibody (ANA) analysis by indirect immunofluorescence (IIF) (Laboratory of Autoimmunity and Metabolism, IRCCS Humanitas Clinical and Research Center—Rozzano, Milan, Italy).

Patients’ sera were isolated from whole blood through centrifugation at 2000* g* for 15 min, and then stored at −20 °C until use.

ANA were tested by IIF on HEp-2 ANA slides (INOVA Diagnostics, San Diego, CA, USA) using serial dilution of patients’ sera (1:80, 1:160, 1:320, 1:640, 1:1280), followed by AlexaFluor488 AffiniPure F(ab′)2 fragment goat anti-human IgG, Fcγ fragment specific (Jackson Immunoresearch Europe Ltd., Suffolk, UK) as previously described [8]. Images were acquired using the Olympus BX53 Upright fluorescence microscope. IP was performed using ^35^S-methionine-labeled K562 cell extract followed by SDS-PAGE and autoradiography, and by RNA-IP using unlabelled K562 cell extract followed by urea-PAGE and silver staining [32]. MSA were determined using reference sera obtained from the Autoantibody Standardization Committee (www.autoab.org) and from internal controls.

Candidates for anti-NXP2 sera were tested by IP-Western blot (IP-WB) based on IP of a 140-kD protein [8, 32]. In detail, 50 µL of candidate sera was cross-linked with protein-A Sepharose beads and then immunoprecipitated with cell extract from 10^7^ K562 cells. Proteins were then fractionated by 8% SDS-PAGE and transferred to a nitrocellulose filter, probed with 2 μg/mL of anti-MORC3 rabbit polyclonal antibody (Novus biological, Centennial, USA) for NXP-2, followed by peroxidase affinity pure goat-anti-rabbit IgG, F(Ab′)2 fragment specific (1:2000 dilution) (Jackson Immunoresearch Europe Ltd., Suffolk, UK) and developed using Immobilon Western Chemiluminescent HRP substrate (Millipore, Darmstadt, Germany).

### Statistical Analysis

Categorical variables were expressed as number or percentage, continuous variables as median and interquartile range (IQR). Comparisons between groups were performed by chi-square test, Fisher’s exact test, Student’s *t* test and Mann–Whitney test when appropriate. A logistic regression model was built to identify clinical manifestations independently associated with NXP2+ with the inclusion of the variables that were significant at univariate analysis. A *p*-value < 0.05 was considered statistically significant. The odd ratio (OR) with 95% confidence interval (95% CI) was calculated. Survival from cancer onset was estimated using Kaplan–Meier method and differences between groups were compared using the log-rank test. The GraphPad4 version was used for statistical analysis. The study was approved by the Ethical Committee of the leading centre (ASST Spedali Civili of Brescia, NP3511).

## Results

Anti-NXP2 antibodies were found in 61 IIM patients, 42 females and 19 males, with a median age at disease onset of 46 years (IQR 28.7–59.2), and a median follow-up of 26 months (IQR 12–120). Demographic data are reported in Table 1: DM was the most common diagnosis, occurring in 42 cases, followed by PM (11 cases), IBM (4 cases), ASS, OM and IMNM in one case, each. Most of the patients were Caucasian (95%). Clinical data were available for 60 patients.

**Table 1:** Demographic data on 61 NXP2+ , detected by LB

	**LB+ NXP2+ ** **n. 61 (%)**
Age at diagnosis, years, median (IQR)	46 (28–59.7)
Follow-up, months, median (IQR)	26 (12–120)
F/M ratio	42/19 (2.1:1)
Caucasian	57 (95)
Number of deaths at the end of follow-up	1/60* (1.6)
Polymyositis (PM)	11/60* (18.3)
Dermatomyositis (DM)	42/60* (70)
Anti-synthetases syndrome (ASS)	1/60* (1.6)
Inclusion body myositis (IBM)	4/60* (6.5)
Necrotizing myositis (IMNM)	1/60* (1.6)
Overlap myositis (OM)	1/60* (1.6)

Data are expressed as median and interquartile range (IQR) or frequencies with percentages (%)

^*^Clinical data are available for 60 patients

### Comparison Between NXP2+ by LB and 211 NXP2−

The comparison between 60 NXP2+ patients, with available clinical data, by LB analysis and 211 NXP2− adult patients is shown in Table 2. NXP2+ were younger at disease diagnosis (median 46 vs 57 years, *p* = 0.0014) with a shorter follow-up (median 25 vs 84 months, *p* = 0.009). They showed a higher frequency of DM (68.3% vs 29.6%, *p* < 0.0001; OR 5.1, 95% CI 2.8–9.6) and IBM (6.7% vs 0.9%, *p* = 0.023, OR 7.4; 95% CI 1.3–41.8) whilst ASS was more frequently detected in NXP2− (*p* < 0.0001, OR 0.059; 95% CI 0.008–0.45). Even with the exclusion of the 6 patients with juvenile onset, NXP2+ remained younger at disease diagnosis (median 48 vs 57 years, *p* = 0.0036) but without differences in the follow-up duration (median 30 vs 84, *p* = NS).

**Table 2:** Demographic and clinical features of 60 NXP2+ by LB and 211 NXP−

	**LB NXP2+** **n.60 (%)**	**LB NXP2−** **n. 211 (%)**	***p* value (OR, 95% CI)**
**Demographic features**			
Age at diagnosis, years, median (IQR)	46 (28.7–59.2)	57 (41–66)	0.0014
Follow-up, months, median (IQR)	25 (11.5–115)	84 (30–144)	0.009
DM	42 (70)	62 (29.4)	<0.0001 (5.60, 3–10.3)
PM	11 (18.3)	64 (30.3)	0.07
IBM	4 (6.7)	2 (0.9)	0.023 (7.4, 1.3–41.8)
ASS	1 (1.6)	47 (22.3)	<0.0001 (0.059, 0.008–0.45)
OM	1 (1.6)	7 (3.3)	0.7
IMNM	1 (1.6)	0	0.22
**Clinical features**			
Skin rash	38 (65.5)	78 (37.5)	0.001 (2.88, 1.58–5.22)
Facial rash	29(50)	63 (30)	0.0013 (2.18, 1.2–3.9)
Heliotrope rash	33 (56.9)	55 (26.3)	<0.0001 (3.4, 1.88–6.2)
Gottron’s papules	18 (31.5)	55 (26.3)	0.6
Periorbital oedema	11 (18.6)	18 (8.6)	0.055 (2.38, 1–5.37)
Peripheral oedema	4 (6.68)	15 (7.1)	1
Fever	16 (27.6)	71 (34)	0.34
Fatigue	48 (82.7)	155 (74.5)	0.49
Periungual telangiectasia	9 (15.2)	33 (15.9)	1
Cutaneous ulcerations	7 (11.8)	28 (13.6)	0.8
Calcinosis	10 (17.2)	13(6.2)	0.017 (3, 1.25–7.27)
Myositis	54 (91.5)	167 (79.9)	0.08
Mechanic’s hands	1 (1.7)	43 (20.57)	<0.0001 (0.06, 0.009–0.48)
Sclerodactyly	1 (1.7)	40 (19)	<0.0001 (0.07, 0.01–0.53)
Puffy hands	1(1.7)	28 (13.4)	0.008 (0.11, 0.015–0.8)
Raynaud’s phenomenon	12 (20.3)	109 (52.1)	<0.0001 (0.22, 0.115–0.46)
Arthritis	11 (18.6)	72 (34.4)	0.018 (0.42, 0.2–0.8)
Dyspnoea	13 (22)	109 (52.1)	<0.0001 (0.25, 0.13–0.49)
Dysphagia	24 (40.6)	61 (29.18)	0.45
ILD	8 (13.8)	98 (46.8)	0.0001 (0.21, 0.097–0.48)
Myocarditis	2 (3.4)	8 (3.98)	1
Scleroderma pattern by NVC*	7/25 (28)	45/110 (40.9)	0.26
Anytime cancer	7/59 (11.8)	40/190 (21.2)	0.13
CAM	3/59 (5)	20/190 (10.5)	0.3

Data are expressed as median and interquartile range (IQR) or frequencies with percentages (%)

*ASS* anti-synthetase syndrome, *CAM* cancer-associated myositis, *DM* dermatomyositis, *IBM* inclusion body myositis, *ILD* interstitial lung disease, *IMNM* necrotizing autoimmune myositis, *NVC* nailfold videocapillaroscopy, *OM* overlap myositis, *PM* polymyositis

^*^Data was available for 25 NXP2+ and 110 NXP2− patients, respectively

Concerning clinical data, NXP2+ cases more frequently showed manifestations typical of DM as facial (*p* = 0.0013; OR 2.18, 95% CI 1.2–3.9) and heliotrope rash (*p* < 0.0001; OR 3.4, 95% CI 1.88–6.2), periorbital oedema (*p* = 0.055; OR 2.38, 95% CI 1–5.37) and calcinosis (*p* = 0.017; OR 3, 95% CI 1.25–7.27). Symptoms associated with ASS or OM were more rarely described in NXP2+ , namely dyspnoea (*p* < 0.0001; OR 0.25, 95% CI 0.13–0.49), ILD (*p* = 0.0001; OR 0.21, 95% CI 0.097–0.48), mechanic hands (*p* < 0.0001; OR 0.06, 95% CI 0.009–0.48), sclerodactyly (*p* < 0.0001; OR 0.07, 95% CI 0.01–0.53), puffy hands (*p* = 0.008; OR 0.11, 95% CI 0.015–0.8), Raynaud’s phenomenon (*p* < 0.0001; OR 0.22, 95% CI 0.11–0.46) and arthritis (*p* = 0.018; OR 0.42, 95% CI 0.2–0.8) as reported in Table 2.

### Comparison Between NXP2+ by LB and IP and 211 NXP2− 

Serum from 52 NXP2+ by LB was available for testing by IP: anti-NXP2 antibodies were confirmed in 31 sera (60%), with the following diagnosis: DM 27 (87%), PM 3 (9.6%) and IBM 1 case (3.2%).

Comparison between LB+/IP+ NXP2+ and 211 NXP2− is reported in Table 3. Whilst the same previously reported associations were confirmed, with greater significance, LB+/IP+ NXP2+ showed more frequently dysphagia (*p* = 0.022; OR 2.5, 95% CI 1.2–5.56) and myositis (*p* = 0.0022, OR 15.8, 95% CI 0.94–263.6) compared with NXP2− group. By contrast, neither the association between anti-NXP2 antibodies and IBM diagnosis nor the shorter length of follow-up in NXP2+ between the two groups was confirmed.

**Table 3:** Demographic and clinical features of 31 NXP2+ patients by line blot and IP (LB+/IP+) and 211 NXP2− patients

	**LB+/IP+ NXP2+ ** **n. 31 (%)**	**LB NXP2−** **n. 211 (%)**	***p* value (OR, 95% CI)**
**Demographic features**			
Age at diagnosis, years, median (IQR)	38 (18.5–56.5)	57 (41–66)	<0.0001
Follow-up, months, median (IQR)	30 (10–120)	84 (30–144)	0.3
DM	27 (83.3)	62 (29.4)	<0.0001 (16.22, 5.4–48.3)
PM	3 (9.6)	64 (30.3)	0.017 (0.24, 0.07–0.84)
IBM	1 (3.3)	2 (0.9)	0.338
ASS	0 (0)	47 (22.3)	0.001
OM	0 (0)	7 (3.3)	0.6
**Clinical features**			
Any type rash	24 (77.4)	78 (37.5)	<0.0001 (5.7, 2.3–13.8)
Facial rash	22 (70.9)	63 (30)	<0.0001 (5.7, 2.48–13)
Heliotrope rash	23 (74.2)	55 (26.3)	<0.0001 (8, 3.4–19)
Gottron’s papules	11 (35.5)	55 (26.3)	0.28
Periorbital oedema	7 (22.5)	18 (8.6)	0.027 (3, 1.17–8.17)
Peripheral oedema	4 (12.9)	15 (7.1)	0.28
Fever	10 (32.3)	71 (34)	1
Fatigue	25 (80.7)	155 (74.5)	0.65
Periungual telangiectasias	4 (12.9)	33 (15.9)	0.74
Cutaneous ulcerations	4 (12.9)	28 (13.6)	1
Calcinosis	7 (22.2)	13 (6.2)	0.007 (4.39, 1.59–12)
Myositis	31 (100)	167 (79.9)	0.002 (15.8, 0.94–263.6)
Mechanic’s hands	0 (0)	43 (20.57)	0.002 (0.06, 0.003–1.02)
Sclerodactyly	0 (0)	40 (19)	0.004 (0.06, 0.004–1.12)
Puffy hands	0 (0)	28 (13.4)	0.031 (0.10, 0.006–1.71)
Raynaud’s phenomenon	6 (19.4)	109 (52.1)	0.001 (0.22, 0.087–0.56)
Arthritis	5 (16)	72 (34.4)	0.062 (0.36, 0.13–0.99)
Dyspnoea	7 (22.6)	109 (52.1)	0.002 (0.26, 0.11–0.64)
Dysphagia	19 (52.8)	61 (29.18)	0.022 (2.5, 1.2–5.56)
ILD	1 (3.3)	98 (46.8)	<0.0001 (0.038, 0.005–0.28)
Myocarditis	2 (6.4)	8 (3.98)	0.622
Scleroderma pattern at NVC*	4/20 (20)	45/110 (40.9)	0.08
Anytime cancer	3 (9.7)	40/190 (21.5)	0.21
CAM	2 (6.4)	20/190 (10.5)	0.74

Data are expressed as median and interquartile range (IQR) or frequencies with percentages (%)

*ASS* anti-synthetase syndrome, *CAM* cancer-associated myositis, *DM* dermatomyositis, *IBM* inclusion body myositis, *ILD* interstitial lung disease, *IMNM* necrotizing autoimmune myositis, *NVC* nailfold videocapillaroscopy, *OM* overlap myositis, *PM* polymyositis

^*^Data was available for 20 NXP2+ and 110 NXP2− patients, respectively

### Cancer Association

Both anytime cancer and CAM myositis were more rarely diagnosed in LB NXP2+ compared with NXP2− , without any significant difference (Table 2). These results did not change with the exclusion of the 6 cases with a juvenile onset: anytime cancer occurred in 6 LB NXP2+ with an adult onset (6/53, 11.3% vs 40/190, 21.2%; *p* = 0.11), and CAM in 3 adults NXP2+ (3/53, 5.7% vs 20/190, 10.5%; *p* = 0.42).

Limiting the analysis to LB+/IP+ NXP2+ group (Table 3), 3 patients presented an anytime cancer, with 2 cases of CAM (a melanoma skin cancer diagnosed 7 months before IIM diagnosis, and a multicentric ductal carcinoma breast cancer found simultaneously at the diagnosis of IIM). Moreover, the exclusion of the 4 patients with juvenile onset did not change the results: anytime cancer occurred in 3 adult NXP2+ (3/27, 11.1% vs 40/190, 21.5%; *p* = 0.30) and CAM in 2 (2/27, 7.4% vs 20/190, 10.5%; *p* = 1). Again, even if a lower cancer prevalence in NXP2+ occurred, this was not significant.

Anti-NXP2 antibodies were not associated with concomitant cancer development, either when positive by LB alone or by both IP and LB methods, as shown by survival analysis (Fig. 1 Supplemental).

### Multivariate Analysis

Multivariate analysis, considering only 31 NXP2+ LB+/IP+ and 211 NXP2− cases, confirms that anti-NXP2 antibodies are associated with DM (*p* = 0.04; OR 6.17, 95% CI 1.07–35) whilst a negative association was confirmed for ILD (*p* = 0.048; OR 0.08, 95% CI 0.007–0.9).

A multivariate analysis of all 60 NXP2+ (detected by LB only) does not confirm the association with DM.

### Analysis of Discordant Sera (LB Positive, IP Negative)

As reported above, 21 NXP2+ sera by LB were not confirmed by IP. These discordant patients showed the same clinical features of 211 NXP2-negative patients; in particular, no differences were found regarding features associated with NXP2 clinical phenotype (i.e. DM rash, calcinosis, dysphagia) (Table 1 supplemental material).

Comparing 21 LB+/IP− and 31 LB+/IP+ NXP2+ , multiple autoantibodies (including MSA and MAA) were more frequent in discordant sera (*p* = 0.0019), as well as multiple MSA (*p* = 0.013), as shown in Table 4.

**Table 4:** Comparison between 21 discordant sera and 31 NXP2+ sera by LB and IP

	**LB+/IP−NXP2+ ** **n. 21 (%)**	**LB+/IP+NXP2+ ** **n. 31 (%)**	***p* value (OR, 95% CI)**
Female	16 (76.2)	21 (67.7)	0.55
DM	10 (50)	27 (87)	0.009 (0.148, 0.038–0.58)
Multiple autoantibodies by LB	8/21 (38)	3 (9.67)	0.019 (5.7, 1.3–25)
Multiple MSA by LB	6 (28.6)	1 (3.22)	0.013 (12, 1.3–108)
IIF multiple nuclear dot pattern	0	11 (35.4)	0.0035 (˂0.0001, ˂0.0001–0.379)

Data are expressed as frequencies with percentages (%)

*DM* dermatomyositis, *IIF* indirect immunofluorescence, *MSA* myositis specific autoantibodies

In particular, amongst the 21 discordant sera, LB assay found the presence of the following concomitant MSA/MAA: anti-Ro (4 sera), anti-SRP (3 sera), anti-Mi-2 (2 sera), anti-Ku (2 sera), anti-MDA5 (1 serum) and anti-TIF1gamma (1 serum). Otherwise, only one NXP2 LB+/IP+ serum presented another MSA (anti-TIF1gamma) by LB, a specificity that was not confirmed by IP assay. The clinical diagnosis of discordant sera was represented by DM (11 cases), PM (6 cases), IBM (1 case), ASS (1 case) and IMNM (1 case). No relationship was found between clinical diagnoses and autoantibodies’ profile, analysed by LB (Table 5).

**Table 5:** Autoantibody profile and clinical data of 21 discordant sera studied by LB and IP

	**IP results**	**LB results**	**ANA pattern IIF**	**Clinical diagnosis**
1	Mi-2, TIF1α	NXP2, Mi-2	Homogeneous	DM
2	Negative	NXP2	Fine speckled	PM
3	Negative	NXP2	Fine speckled	PM
4	Negative	NXP2	Fine speckled	PM
5	Negative	NXP2	Large speckled	DM
6	Negative	NXP2+ SRP+ Ku	Speckled	IMNM
7	Negative	NXP2	Speckled	PM
8	Negative	NXP2	Homogeneous + speckled	DM
9	Negative	NXP2	Nucleolar + speckled	DM
10	Negative	NXP2	Cytoplasmic	DM
11	Negative	NXP2	Cytoplasmic	DM
12	Negative	NXP2	Negative	DM
13	TIF1γ/α	NXP2+ MDA5 (borderline)	Speckled	DM (probably CAM)
14	Negative	NXP2+ TIF1γ+ Ro	Negative	DM
15	OJ	NXP2+ Ku+ Ro	Negative	OM
16	Ro60	NXP2+ Ro52	Positive	PM
17	Negative	NXP2	Positive	DM
18	Negative	NXP2	Homogenous	IBM
19	Negative	NXP2	Negative	PM
20	EJ, Ro	NXP2, SRP	Speckled	DM
21	EJ	NXP2, Ro, Mi2, SRP	Negative	ASS

*ASS* anti-synthetase syndrome, *CAM* cancer-associated myositis, *DM* dermatomyositis, *IBM* inclusion body myositis, *IMNM* necrotizing autoimmune myositis, *OM* overlap myositis, *PM* polymyositis

IIF on HEp-2 cells revealed a positivity in all the 31 double-positive (LB+/IP+) sera with the following patterns: 19 speckled (61.3%), 7 multiple nuclear dots (22.5%), 3 speckled and multiple nuclear dots (9.7%), 1 speckled with cytoplasm positive speckled (3.2%) and 1 multiple nuclear dots and cytoplasm positive (3.2%). Globally, a multiple nuclear dot pattern that may be referred as the PML staining typical for anti-NXP2-positive patients was found in 11 LB+/IP+ sera, whereas this pattern was not found in any of the 21 LB+/IP− sera (*p* = 0.0035).

## Discussion

Anti-NXP2 antibodies, also known as anti-MJ, were first described as a marker of juvenile DM, and later also found in adult onset DM. A peculiar clinical spectrum, associated with anti-NXP2 antibodies, characterized by DM rash, in particular Gottron’s papules, heliotropic rash, peripheral oedema and periungual telangiectasias [2, 6, 8, 10–12], dysphagia and calcinosis has been described [6, 10, 11, 33–35].

These clinical associations are (mostly) based on the detection of anti-NXP2 by IP [8, 10, 11]. However, the availability of IP assay is confined to few research centres, whereas, nowadays, laboratories are widely using commercially available immunoassays, such as LB, for the detection for MSA or MAA.

This large Italian multicentre study analysed the clinical spectrum associated with anti-NXP2 antibodies detected by LB, confirming previously described associations, namely skin rash, facial rash, heliotropic rash, periorbital oedema and calcinosis [6, 10, 11]. In contrast, symptoms associated with overlap myositis or ASS were not found to be associated with NXP2 antibodies, including ILD, thus confirming previous data [6].

When detected only by LB, we found anti-NXP2 antibodies significantly associated with IBM: this unusual observation was not reported in other case series, reviewed in a recent meta-analysis [6], and was not confirmed when anti-NXP2 antibodies were searched by IP. In fact, double-positive NXP2 patients were mostly affected by DM. IBM is a different type of IIM, with peculiar clinical and histological features, recently characterized by a new autoantibody specificity, known as anti-cN1A [36].

Anti-NXP2 antibodies were confirmed by IP in 31 on 52 available sera (60%). When the 31 double-positive patients were compared with IIM NXP2− patients, the previously described clinical associations were confirmed with higher significance, and dysphagia and myositis were also added as associated with anti-NXP2 antibodies. Most of the papers reported a strict correlation between anti-NXP2 antibodies and dysphagia [2, 6, 10, 11, 13, 33], whilst the occurrence of myositis in anti-NXP2-positive patients is less evident in different cohorts. Nevertheless, a severe muscle weakness with myalgia [6, 11], with higher levels of CK [11] or necrotizing histological features [37] have been described in some NXP2+ case series.

Multivariate analysis, performed on 31 double-positive sera, confirmed the association between NXP2 and DM, and a negative association with ILD was also assessed.

The description of a possible link between anti-NXP2 antibodies and cancer was reported by some authors [13, 15, 38], but two recent meta-analyses did not confirm this hypothesis [7, 12]. In 2012, Ichimura and colleagues firstly reported the association between CAM and anti-NXP2+ IIM on 8 patients, 3 of whom had cancer within 3 years from diagnosis [38]. Fiorentino and colleagues in 2013 confirmed this association in 37 anti-NXP2-positive patients with DM: at multivariate analysis, cancer was associated with either NXP2 or TIF1gamma antibodies, older age, and male sex [15]. Finally, in 2017, Albayda reported an increased risk for cancer in a cohort of 56 NXP2+ DM patients compared with the general population (3.68-fold increased risk), whereas no differences were found between NXP2+ and NXP2− IIM cases [13]. No association with increased risk of cancer was otherwise reported in several other multicentric studies [2, 8, 39]. In the present study we also did not confirm an increased risk of cancer associated with anti-NXP2 antibodies, although we did not perform a direct comparison with the general population. These discrepant data could be due to the low number of patients considered and/or the different ethnic background of different cohorts [15, 38, present study]. Although MORC3 protein is known to be involved in cellular senescence, p53 recruitment [40] and oncogenesis [41], the clear demonstration of the oncogenic potential of anti-NXP2 antibodies was not reported, so far, in contrast to what has been described for anti-TIF1gamma [42] or anti-RNAPol-III antibodies, specific for SSc [43].

The reliability between the different methods of detection of MSA and/or MAA remains a topic of discussion. Immunoprecipitation has historically been used to identify MSA and MAA, but it is an impractical method for widespread diagnostic use. Conversely, commercial multiplex assay represents an easier, low-cost method that can simplify the detection of these autoantibodies in clinical practice.

In our cohort the gold standard IP did not confirm the result of the multiplex assay technique in nearly 40% of cases, and moreover, these patients did not present clinical features included in the clinical spectrum of NXP2 antibody. It has been already demonstrated that multiple testing, whereas increasing efficiency could also increase false-positive results. Several reports described how the agreement between commercial assay and IP may be influenced by the rarity of the autoantibody or by the antigen specificity and no conclusive data are currently available [16–26, 44]. In 2019, Fiorentino et al. compared results from LB and IP in DM patients, and only a fair concordance was found for NXP2 specificity [26] differently from the moderate concordance shown by a previous study [17]. Otherwise, in a recent paper of Tansley et al. a high agreement was found between LB, dot blot and IP [44]. Linked to the problem of false-positive results is the frequency of double- or multiple-positive MSA. In literature, the presence of a concomitant MSA is considered a rare event, with a prevalence reported between 0 and 0.2% [2, 17] in studies using IP. When using commercial assays, the prevalence of a co-existing MSA is much higher: in a previous Italian work, the prevalence using LB was above 15% [17], more recently reported as 6% in an English study [25]. In our analysis we have found that discordant sera more frequently presented an associated MSA compared with double-positive NXP2, confirming the idea that when multiple MSAs are found in commercial testing, results should be carefully managed. In fact, even if multiple MSAs appear to be associated with IIM, the clinical subset cannot be clearly defined.

NXP2 antibody is not associated with a unique IIF pattern: in a previous study the 60% of NXP2 sera positive with IP displayed a nuclear dot pattern consistent with the presence of PML bodies [9]. In the present study, this pattern was found only in the 35% of double-positive patients, but in none of the sera not confirmed by IP. Therefore, even if we can state that IIF could not be considered as a confirmation method, the presence of multiple nuclear dot pattern could help to discriminate true NXP2 patients [45].

The present study has some limitations. First, serum was not available neither for all NXP2 patients positive with commercial assay nor for the control group; therefore not all the patients were centrally tested with IP, thus limiting the analysis of concordance between assays. Moreover, only a single sample was available, not allowing longitudinal analysis. Recently, a longitudinal evaluation of 14 NXP2 patients confirmed how this MSA, similar to others, can fluctuate along with changes in disease activity, suggesting a possible role as a biomarker for monitoring during follow-up [46]. Finally, data obtained with the LB were not compared with other commercial assay; however, it has been recently demonstrated a good agreement between the results of LB and dot blot assay regarding NXP2 [20].

The main strengths are the number of clinical cases from different Italian centres and the prospective evaluation of sera collected by IP and IIF on HEp-2 cells.

In conclusion, double-positive sera for LB and IP describe a clinical spectrum characteristically associated with anti-NXP2 antibodies. Whilst we recognize the importance of the routine use of multiple testing to diagnose patients affected by IIM, the only use of commercial LB assay seems to not adequately identify anti-NXP2-positive patients falling in the NXP2 classical clinical spectrum.

## Supplementary Information

Below is the link to the electronic supplementary material.

Supplementary file1 (PDF 127 KB)

Supplementary file2 (PDF 77 KB)

## Data Availability

The datasets used and/or analysed during the current study are available from the corresponding author on reasonable request.
