# Supplementary material for: An Italian Multicenter Study on Anti-NXP2 Antibodies: Clinical and Serological Associations
Source: Clin Rev Allergy Immunol. 2022 Jan 29;63(2):240–50. doi: 10.1007/s12016-021-08920-y (PMC9464148; doi:10.1007/s12016-021-08920-y)
Supplement: Supplementary file 1 — Supplementary file1 (PDF 127 KB) [file 12016_2021_8920_MOESM1_ESM.pdf]

Table 1 supplemental material: comparison between 21 discordant sera (LB+/IP-) and 211 NXP2 negative patients

	LB+/IP- n.21 (%)	LB NXP2 - n. 211 (%)	P value (OR;95%CI)
**DEMOGRAPHIC FEATURES**			
Age at diagnosis, years, median (IQR)	59 (44.5-66.5)	57 (41-66)	0.45
Follow-up, months, median (IQR)	24 (10.7-69)	84 (30-144)	0.002
DM	10 (47.6)	62 (29.4)	0.13
PM	6 (28.5)	64 (30.3)	1
IBM	1 (4.8)	2 (0.9)	1
ASS	1 (4.8)	47 (22.3)	0.10
OM	1 (4.8)	7 (3.3)	0.53
IMNM	1 (4.8)	0	0.09
**CLINICAL FEATURES**			
Skin rash	9 (42.8)	78 (37.5)	0.76
Facial rash	4 (19)	63 (30)	0.43
Heliotrope rash	3 (14.3)	55 (26.3)	0.31
Gottron's papules	2 (9.5)	55 (26.3)	0.11
Periorbital edema	2 (9.5)	18 (8.6)	0.69
Peripheral edema	0	15 (7.1)	0.37
Fever	4 (19)	71 (34)	0.22
Fatigue	16 (76.2)	155 (74.5)	0.99
Periungual telangiectasia	4 (19)	33 (15.9)	0.75
Cutaneous ulcerations	1 (4.8)	28 (13.6)	0.48
Calcinosis	2 (9.5)	13 (6.2)	0.63
Myositis	15 (71.4)	167 (79.9)	0.41
Mechanic's hands	1 (4.8)	43 (20.6)	0.13
Sclerodactyly	1 (4.8)	40 (19)	0.13
Puffy hands	0	28 (13.4)	0.14
Raynaud's phenomenon	6 (28.6)	109 (52.1)	0.07
Arthritis	3 (14.3)	72 (34.4)	0.10
Dyspnea	6 (28.6)	109 (52.1)	0.07
Dysphagia	5 (23.8)	61 (29.18)	0.80
ILD	5 (23.8)	98 (46.8)	0.08
Myocarditis	0	8 (3.98)	1
Scleroderma Pattern by NVC*	3/7 (42.8)	45/110 (40.9)	1
Anytime Cancer	3 (14.3)	40/190 (21.5)	0.58
Cancer associated myositis	1 (4.8)	20/190 (10.5)	0.70

Data are expressed as median and interquartile range (IQR) or frequencies with percentages (%). ASS= antisynthetase syndrome; CAM= cancer associated myositis; DM= dermatomyositis; IBM= inclusion body myositis; ILD= interstitial lung disease; IMNM= necrotizing autoimmune myositis; OM= overlap myositis; NVC* =nailfold videocapillaroscopy (NVC) data was available only for 7 NXP2+ discordant LB+/IP- and 110 NXP2- patients, respectively; PM=polymyositis.
