# Supplementary figures and images for: An Italian Multicenter Study on Anti-NXP2 Antibodies: Clinical and Serological Associations
Source: Clin Rev Allergy Immunol. 2022 Jan 29;63(2):240–50. doi: 10.1007/s12016-021-08920-y (PMC9464148; doi:10.1007/s12016-021-08920-y)

Supplementary Figure 1

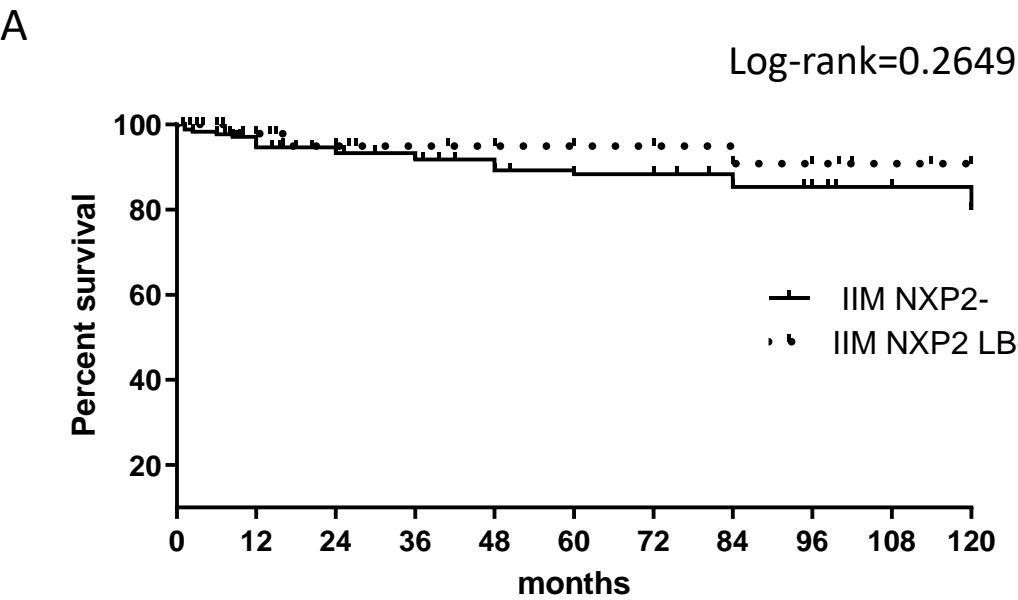

| Patients at risk | 0   | 12  | 24  | 36  | 48  | 60  | 72 | 84 | 96 | 108 | 120 |
|------------------|-----|-----|-----|-----|-----|-----|----|----|----|-----|-----|
| NXP2-            | 175 | 158 | 137 | 126 | 107 | 102 | 93 | 88 | 78 | 68  | 63  |
| NXP2 LB+         | 56  | 42  | 33  | 28  | 26  | 25  | 24 | 23 | 22 | 19  | 17  |

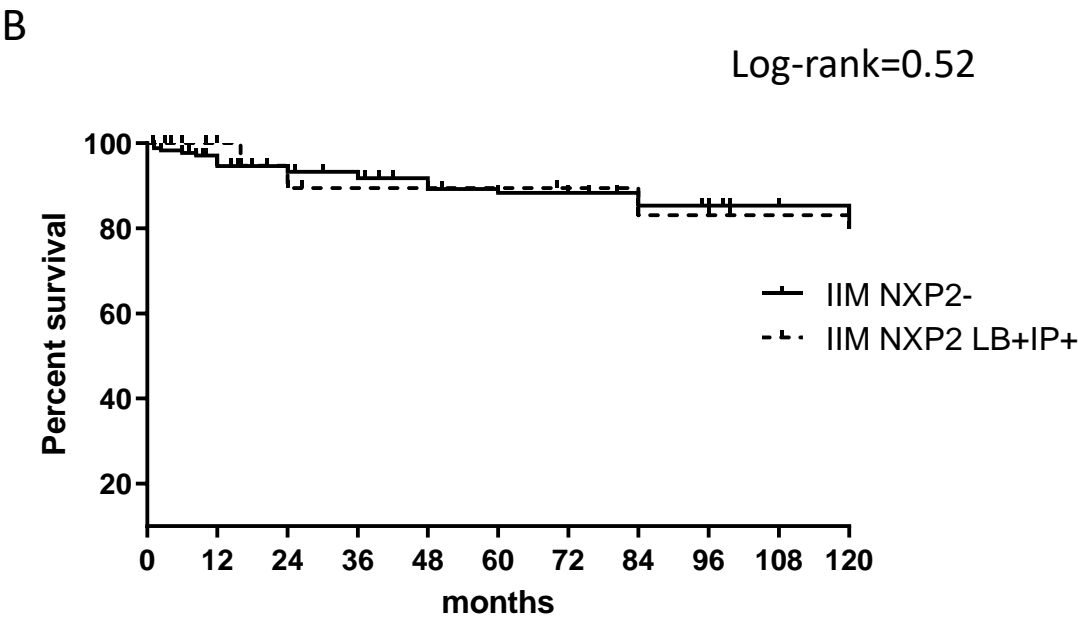

| Patients at risk | 0   | 12  | 24  | 36  | 48  | 60  | 72 | 84 | 96 | 108 | 120 |
|------------------|-----|-----|-----|-----|-----|-----|----|----|----|-----|-----|
| NXP2-            | 175 | 158 | 137 | 126 | 107 | 102 | 93 | 88 | 78 | 68  | 63  |
| NXP2 LB+/IP+     | 28  | 22  | 18  | 16  | 16  | 16  | 15 | 14 | 13 | 12  | 10  |

Supplement: Supplementary file 2 — Supplementary file2 (PDF 77 KB) [file 12016_2021_8920_MOESM2_ESM.pdf]
